# Memory for emotional information and alexithymia A systematic review

**DOI:** 10.1590/1980-57642018dn13-010003

**Published:** 2019

**Authors:** Leonardo T. Apgáua, Antônio Jaeger

**Affiliations:** 1 Universidade Federal de Minas Gerais, Brazil.; 2 Department of Psychology, Universidade Federal de Minas Gerais, Brazil.; 3 Instituto Nacional de Ciência e Tecnologia sobre Comportamento, Cognição e Ensino, São Carlos, SP, Brazil.

**Keywords:** alexithymia, emotion, memory, recall, priming, alexitimia, emoção, memória, recordação, priming

## Abstract

Alexithymia is a deficit in the recognition, expression and regulation of emotions, which has the following features: difficulty in identifying or describing feelings, difficulty distinguishing between feelings and bodily sensations, stringent imaginal processes, and externally oriented cognitive style. This personality trait is associated with many psychiatric and psychosomatic disorders, as well as with risky behaviors.

**Objective:**

To investigate whether this trait is also associated with reduced memory for emotional information.

**Methods:**

A review of articles investigating the possible damage caused by alexithymia to implicit and explicit memory for emotional information was conducted.

**Results:**

Although the studies concerning implicit memory presented divergent results, most studies on explicit memory suggested a deficit for emotional information retention in high-alexithymia individuals.

**Conclusion:**

The reviewed data support the notion that the typical increase in episodic memory for emotional information is reduced in high-alexithymia individuals.

## RESUMO.

A alexitimia é um déficit no reconhecimento, expressão e regulação das emoções, que possui as seguintes características: dificuldade em identificar sentimentos, dificuldade em descrever sentimentos, dificuldade em distinguir sentimentos e sensações corporais, processos de imaginação dificultados e estilo de cognição externamente orientado. Este traço de personalidade está associado a muitos transtornos psiquiátricos e psicossomáticos, bem como a comportamentos de risco aumentados.

**Objetivo:**

Investigar se este traço também está associado a redução de memória para informações emocionais.

**Métodos:**

Artigos que investigam os possíveis danos causados ​​pela alexitimia na memória implícita e explícita para informações emocionais foram revisados.

**Resultados:**

Embora os estudos sobre memória implícita tenham apresentado resultados divergentes, a maioria dos estudos sobre memória explícita sugeriu déficits na retenção de informação emocional em indivíduos com altos índices de alexitimia.

**Conclusion:**

Os dados revisados ​​sugerem que o aumento típico na memória episódica para informações emocionais é reduzido em indivíduos com alta alexitimia.

A lexithymia involves deficits in the recognition, expression and regulation of emotions, as well as difficulties in identifying and describing emotions, distinguishing emotions from other bodily sensations, impoverished imaginal processes, and externally oriented cognitive style. These features involve reduced awareness of one’s own emotions and a focus of attention on practical details of events instead of on their emotional features. The psychological construct of alexithymia was first developed by Peter Sifneos and its etymology derives from Greek, meaning “no words for emotions” (a = lack, lexis = word, thymos = emotion or humor;).^1^ The trait of alexithymia is relatively stable among different degrees of emotion, stress and psychopathology.^2^

Individuals with alexithymia experience confusion while trying to identify their own feelings and show difficulty to consciously relate them to their memories.^3^ These characteristics are associated with a constant inability to properly regulate affect, to consciously disengage attention from unwanted information, and to efficiently convey one’s affective state to others.^4^^-^ 6 High-alexithymia individuals are known to exhibit less facial expression than people in general, even while debating relevant personal events, both positive and negative.^7^ When asked how they feel in emotional situations they tend to use vague expressions (e.g., “I feel sad”), express physical complaints (e.g., “My stomach feels upset”), or demonstrate uncertainty (e.g., “I don’t know”).^8^ Although usually presenting less of a subjective response, the physiological response remains within the normal range,^9^ and may be actually increased in high-alexithymia individuals.^4^

Clinically relevant alexithymia was found in 11.1% of men and 8.9% of women in the general population by Franz et al.^10^ Similar results were also revealed by further studies (e.g. 12.8% of men, 8.2% of women.^11^ Alexithymia is significantly associated with cardiovascular and psychosomatic disorders, as well as with functional gastrointestinal, eating, and panic disorders.^12^ Significant correlations were also found with dissatisfaction in affective relationships,^13^ as well as a tendency toward addictive behaviors, such as substance abuse^14^ and gambling addiction.^15^

Although the etiology of alexithymia has yet to be understood, certain brain structures have been consistently implicated in its manifestation. A reduction in gray matter in the anterior cingulate cortex, anterior insula, orbitofrontal cortex, medial temporal gyrus and superior temporal sulcus has been identified.^16^ Interestingly, the most important difference in gray matter volume between alexithymic and non-alexithymic individuals was found in the anterior cingulate cortex, a region that plays an important role in self-analysis and the perception of a person’s own emotion.^17^^,^ 18 There is also research that points to the relationship between alexithymia and deficits in interhemispheric connectivity,^19^ as well as to diminished activation of the left amygdala during the presentation of visual emotional stimuli, and less response from areas associated with visual encoding of facial expressions.^20^

Thus, because retention of emotional information often involves amygdala activity,^21^ the question arises whether individuals with high alexithymia also have impoverished memory for emotionally charged information. That is, do the deficits in the abilities of these individuals to recognize, express, and regulate emotions affect their capacity to remember emotional past events? In the present article, we sought to answer this question by conducting a review of studies investigating memory for emotional information in individuals with different levels of alexithymia. We conducted an exhaustive search for studies investigating memory and alexithymia, and included studies addressing different explicit and implicit memory constructs, such as episodic memory, autobiographic memory, and priming. Overall, our findings support the notion that high rates of alexithymia diminish the typical increase in explicit memory for emotional information.

## METHODS

We conducted systematic searches on the *Pubmed*, *Web of Science*, and *PsycInfo* databases in January 2019, using the descriptors “alexithymia”, and “emotional”, and “memory”. Further searches were conducted of the reference lists of the articles selected from the databases searches. The selection of the manuscripts for the current systematic review was based on the following inclusion criteria; (1) articles should be published in peer-reviewed scientific journals; (2) articles should report original empirical data; (3) studies should report appropriate data analysis procedures and statistical treatment of the results; (4) studies should investigate memory for emotional information through well-designed memory tests, with a thorough description of the stimuli materials; (5) the type of memory examined should be clearly stated in the report, and the experimental paradigm adopted should be appropriate for the investigation of the specific type of memory studied; (6) studies should assess alexithymia using appropriate scales (e.g., the Toronto Alexithymia Scale); (7) studies should consider the level of alexithymia of the participants in their analysis; (8) there was no restriction regarding the date of publication.

**Figure 1:**
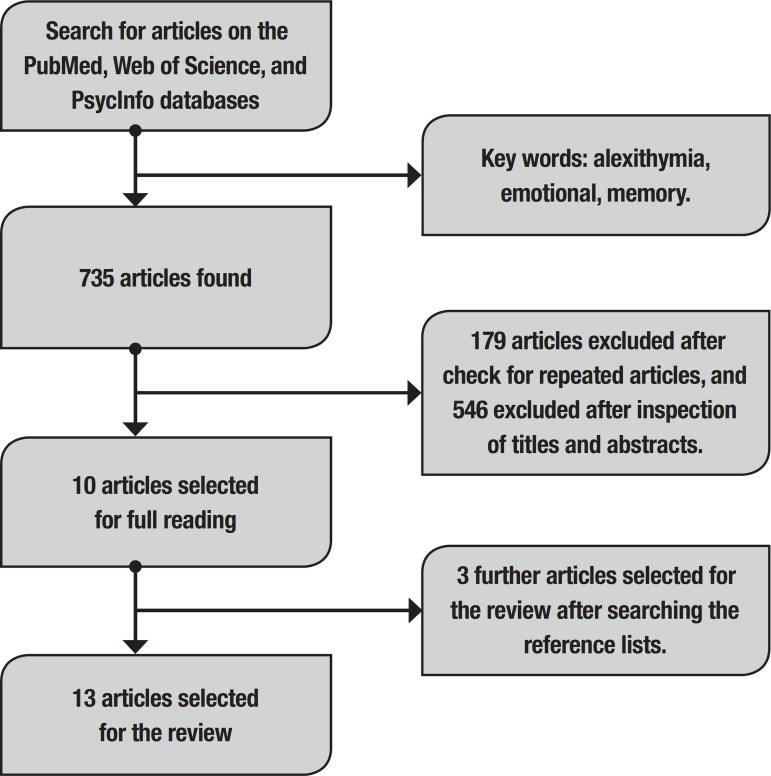
Flowchart describing the steps of the selection of articles for the review.

Initially, 735 articles were retrieved using the keywords in the databases searched (92 were found on the *Web of Science*, 548 in *PubMed*, and 95 in *PsycInfo*). After excluding repeated articles (179 articles), the number of articles decreased to 556. After this, we read each article’s title and abstract (see Figure 1) and excluded 546 articles based on the above inclusion criteria. After this exclusion, only ten articles were retained for the analysis. The searches conducted of their reference lists resulted in the inclusion of three further articles. Thus, in total, 13 articles were selected for the review.

## RESULTS

The results of experiments using implicit memory tests will be presented first, followed by the results of experiments using recognition memory tests, remember/know tests, free recall tests, and autobiographical memory tests. We found four studies using implicit memory tests (priming), two studies using basic recognition memory paradigms, two using a remember/know paradigm, three using free-recall tests, and two using autobiographical memory tests. The type of memory test and the main results of the reviewed studies are described in Table 1. Before addressing these studies, however, it is important to briefly introduce the instrument used to assess alexithymia in all these studies, namely, the Toronto Alexithymia Scale (TAS-20).

**Table 1:** Memory tests adopted by the reviewed studies and their respective main results.

Studies reviewed	Memory test	Main results
Suslow, 1998^30^	Priming	Individuals with heightened difficulty in describing feelings and externally oriented thinking exhibited greater emotional priming effects.
Suslow and Junhanns, 2002^31^	Priming	High-alexithymia individuals exhibited negative priming effects.
Vermeulen et al., 2006^25^	Priming	High-alexithymia individuals showed less priming effects when targets were preceded by angry faces.
Brandt et al. 2015^28^	Priming	High-alexithymia was associated with greater emotional priming when targets were illness-related.
Vermeulen et al. 2010^26^	Recognition	High-alexithymia individuals recognized fewer happy and angry words, and showed decreased recognition for words encoded while angry music was played.
Donges and Suslow, 2015^33^	Recognition	Individuals with greater difficulty in describing feelings showed diminished recognition for faces with expressions of fear and anger.
Luminet et al. 2006^23^	Remember/know	Individuals with greater difficulties in identifying feelings showed diminished recall of positive and negative words.
Vermeulen and Luminet, 2009^24^	Remember/know	Individuals with greater difficulties in identifying feelings exhibited lower memory for emotional words, whereas individuals with greater externally oriented thinking showed greater memory for emotional words.
Suslow et al.^36^	Free recall	Individuals with greater difficulties in identifying feelings showed lower recall of emotional words.
Meltzer and Nielson, 2010^37^	Free recall	High-alexithymia individuals exhibited lower recall of negative words and higher recall of words related to diseases.
Dressaire et al. 2014^27^	Free recall	High-alexithymia individuals exhibited diminished recall of negative words intended to be remembered, and greater recall of negative words intended to be forgotten.
Lundh et al. 2002^40^	Autobiographical	There was no correlation between alexithymia and autobiographical memory.
Muir et al. 2016^29^	Autobiographical	High-alexithymia individuals exhibited a decrease in the typical fading affect bias, showing instead an increase in the forgetting of positive autobiographical memories.

### The Toronto Alexithymia Scale

The TAS-20 questionnaire is the most widely used instrument for assessing alexithymia and was validated on prediction, convergence, discrimination and concomitance criteria,^22^ in spite of cultural differences in more than 19 countries, in both men and women, in clinical and non-clinical samples. It is composed of 20 self-describing propositions that are evaluated by the patient through a Likert scale with a variance of 1 (strongly disagrees) to 5 (strongly agrees). The items are divided equally between three subscales, namely: (a) difficulty identifying feelings (e.g., “When I am upset, I do not know if I am sad, scared or angry”); (b) difficulties describing emotions (e.g., “I find it difficult to describe how I feel about people”); and (c) externally oriented thinking (e.g., “I prefer to talk to people about their daily activities than about their feelings.”). The questionnaire provides a score for the total alexithymia level and specific scores, related to its three subscales: difficulty in identifying feelings (DIF), difficulty in describing feelings (DIF), and externally oriented thinking (EOT).

On the scale, individuals with scores between 20 and 51 points are considered non-alexithymic; with scores in the interval between 52 and 60 points as “possibly alexithymic”; and with scores from 61 up to 100 points as alexithymic with clinical relevance. Of the studies analyzed in the current review, seven comprised groups with high enough alexithymia rates to be considered clinical samples, namely, the studies by Luminet et al.,^23^ Vermeulen and Luminet,^24^ Vermeulen et al.,^25^ Vermeulen et al.,^26^ Dressaire et al.,^27^ Brandt et al.,^28^ and Muir et al.^29^ In the remaining studies, the high-alexithymia group presented mean scores below the range of possible alexithymia (less than 52 points on the TAS-20 - Table 1), with few participants in the clinical range for alexithymia.

### Implicit memory tests

Implicit memory can be assessed by memory tests in which there is no involvement of conscious processes at encoding or retrieval. The priming effect specifically, occurs when a stimulus (e.g., the word “tree”) facilitates the processing of a related stimulus presented shortly thereafter (e.g., “fruit”). The four studies reviewed below examined the effects of priming for emotional stimuli in individuals with different alexithymia scores on the TAS-20.

The first study assessing priming effects for emotional information in alexithymia individuals was reported by Suslow.^30^ In this study, emotional words were shown for 200 ms (primes) followed by a blank screen (100 ms) that preceded the appearance of the target emotional words (shown for 500 ms). Participants were asked to pronounce the target words as quickly and accurately as possible, and to evaluate them as either positive or negative. Priming here corresponded to a facilitation effect in congruent (i.e., when the prime and the target words were from the same emotional category) relative to incongruent trials. Results on this task showed significant positive correlations between the subscale “difficulty describing feelings” and priming effects for negative words, and between the subscale “externally oriented thinking” and priming effects for positive words. That is, individuals with higher scores on these subscales exhibited heightened facilitation in the processing of emotional words preceded by words with equivalent emotions (see Table 1).

The second study assessing priming effects for emotional information according to alexithymia level was reported by Suslow and Junghanns,^31^ and examined priming effects in a lexical decision task (i.e., decide whether a set of letters is a “word” or a “pseudoword”). In their study, neutral and emotional words from the categories love and joy, fear and panic, sorrow and sadness, anger and hatred, as well as “pseudowords” (created by changing one letter of the actual words; e.g., moy, arxiety, housefold) were presented as targets. All words and non-words were primed by sentences describing either emotional or neutral scenarios. While low-alexithymia individuals exhibited the typical congruence/incongruence priming effects, individuals with the highest scores on the TAS-20 showed the opposite effect. That is, they were slower to make the lexical decision when the prime sentences were congruent with the subsequent emotional word and faster when the prime sentences were incongruent with the emotional word. This “negative” priming effect was interpreted by the authors as an indication that high-alexithymia individuals allocate more attentional resources to the emotional content of the stimulus in emotional contexts than low-alexithymia individuals. Note that this finding is inconsistent with the study reviewed above.^30^ Moreover, the following two studies examining emotional priming and alexithymia show divergent results. They used paradigms in which facial expressions were used as primes for emotional target words.

The first study using facial expressions (and emotional words) as primes was reported in Vermeulen et al.^25^ In each trial of the experiments reported by these authors, after the presentation of a word or a face imbued with a specific emotion (100 ms), a target item appeared, and participants were instructed to evaluate them as positive or negative as quickly as possible. In all experiments, high-alexithymia was positively correlated with a significant decrease in the priming effect caused by faces with angry expressions. In other words, high-alexithymia individuals showed less priming effect when items were preceded by angry faces, a result that remained significant after controlling for negative affect, trait anxiety, and depression.

Verbal and facial primes were also used by Brandt et al.^28^ In their study, not only self-reported alexithymia was considered (using the TAS-20), but also observer-rated alexithymia (assessed by the Observer Alexithymia Scale - OAS).^32^ Verbal stimuli were emotionally positive, negative, or neutral words, as well as illness-related words. The priming task comprised all possible combinations of prime-target pairs (verbal-facial, verbal-verbal, facial-facial and facial-verbal), and targets were never neutral. As in Vermeulen et al.,^25^ participants had to evaluate targets as positive or negative as quickly and accurately as possible. After controlling for effects of depressive symptoms, positive and negative affect, high self-reported alexithymia was associated with facilitation effects of positive and negative verbal primes when targets were illness-related. In both conditions, the subscale “difficulty identifying feelings” showed a significant correlation with these facilitation effects. No significant correlations were found between priming effects and observer-rated alexithymia, which was uncorrelated with self-reported alexithymia.

Overall, the results are inconsistent across the studies using implicit memory measures. The first study showed a positive association between high Alexithymia and priming effects for emotional words,^30^ while the second showed the opposite pattern, namely, a “negative” emotional prime for high-alexithymia individuals.^31^ The remaining two studies showed diminished priming effects when angry faces were the primes,^25^ and increased priming effects when targets were illness-related words.^28^ The disparate results from such a small sample of experiments makes it difficult to extract a coherent picture of the relationship between alexithymia and emotional priming. This lack of consistency among the implicit memory studies may indicate that alexithymia interacts more with memory processes involving conscious awareness and cognitive control (see below). In any case, this scenario calls for the development of further research to shed light on the interaction between alexithymia and implicit memory for emotional information.

### Recognition memory tests

The first basic recognition study^26^ examined whether high alexithymia produced lower long-term memory for emotional information by discreetly presenting happy and angry musical pieces during the encoding of happy and angry target words (incidental encoding). At test, individuals with high alexithymia scores recognized fewer happy and angry words than individuals with low alexithymia scores. Furthermore, high alexithymia individuals showed decreased recognition rates than low alexithymia individuals in the angry music condition specifically. These results suggest an overall recognition memory deficit for emotional information in high-alexithymia individuals.

The second recognition memory study investigated memory for emotional faces in undergraduate students with various levels of alexithymia.^33^ In the first phase of the experiment (encoding), photos of faces with expressions of anger, fear, and happiness, were presented among neutral faces. Participants were asked to evaluate whether each face was threatening or non-threatening, and to memorize it for a future test. In the second phase (test), the studied faces were presented again among new faces, and participants judged whether each face had been presented at encoding (“old”) or not (“new”). The authors found that higher TAS-20 scores for the factor “difficulty in describing feelings” was negatively correlated with memory for faces with expressions of fear and anger, suggesting a memory deficit for this type of information in high-alexithymia individuals.

The data reported by these recognition memory studies support the notion that the retrieval of emotional information is reduced in individuals with high levels of alexithymia. The experiments described above, however, focused only on recognition memory. According to the dual-process model framework,^34^ recognition memory tests may involve familiarity processes (i.e., a strength-like, subjective sense of familiarity) or recollection processes (i.e., retrieval of qualitative and contextual information about the event).

A memory test that is typically used to probe whether each memory judgment is engaging familiarity or recollection is the “Remember/Know” paradigm.^35^ In this paradigm, participants are asked to respond with “remember” for items eliciting retrieval of contextual and qualitative details of encoded events, and “know” for items known to be seen in the encoded list, but that do not produce the retrieval of further contextual details of the encoded events. Thus, would recollection versus familiarity processes be differentially impaired in high-alexithymia individuals when the Remember/know procedure was used? This question was addressed by the following two studies.

### Remember/know tests

In the first Remember/know study, Luminet et al.^23^ investigated whether memory for negative and positive valence words was reduced in individuals with high-alexithymia scores. Participants were separated into two groups according to their scores on the TAS-20 (i.e., low and high-alexithymia). The authors also manipulated levels of processing at encoding by conducting a superficial processing task (perceptual) and a deep processing task (semantic). Participants were then unexpectedly asked to recall the studied words, and to indicate whether each word produced a “remember” or “know” state of consciousness. High-alexithymia individuals recalled less positive emotional words than low-alexithymia individuals, for both levels of processing. They also recalled less negative words, but only when the words were from the deep encoding condition. Regarding the remember/know responses, high-alexithymia individuals produced significantly fewer “Remember responses” for emotional words than low alexithymia individuals. For positive words, specifically, the number of “Remember” responses was negatively correlated with participants’ total TAS-20 score (as well as with scores on its three subscales). For negative words, individuals with higher overall scores on the TAS-20, and higher scores on the subscale “difficulty identifying feelings”, also showed fewer “Remember” responses.

The second study using a Remember/know paradigm also manipulated levels of processing^24^ and used emotional words as stimuli. These authors found that scores on the subscale “difficulty identifying feelings” were negatively correlated with memory performance for emotional words. Furthermore, “Remember” responses decreased in all emotional categories as the score in “difficulty identifying feelings” subscale increased. Surprisingly, the construct “externally oriented thinking” was positively correlated with the number of emotional words recognized (especially with “remember responses”), which may explain the lack of correlation for the total TAS-20 score found in this experiment.

Considering these two remember/know studies, high-alexithymia individuals seem to show decreased overall memory for emotional information relative to low-alexithymia individuals, but this difference is apparently more prominent for “remember” than “know” responses. This suggests that alexithymia is more damaging to processes of recollection than to processes of familiarity during the retrieval of emotional information. This issue will be further addressed below by the three studies using free-recall paradigms.

### Free-recall tests

The first study using free recall was reported by Suslow, Kersting and Arolt^36^ and examined the incidental learning of emotional words, considering alexithymia level and depth of processing at encoding. In this experiment, participants evaluated the valence of emotional target words (adjectives) as positive or negative. Each of these targets was preceded by a distractor word (positive, negative, or neutral), shown for 200 milliseconds. Participants were instructed to avoid paying attention to the distractors and to focus on the target words. In unexpected free-recall tests comprising all studied stimuli (including the distractors), high-alexithymia individuals exhibited a significant decrease in memory performance for positive emotional distractors. This impairment was positively correlated with the scores on the subscale “difficulty identifying feelings”. Interestingly, high-alexithymia participants also made significantly more evaluation mistakes than low-alexithymia individuals regarding the valence of emotional target words at encoding.

The second study using free recall was reported by Meltzer and Nielson^37^ and compared memory performances between high and low-alexithymia individuals for emotional, neutral, and illness-related words. Participants initially read words from four categories: highly arousing positive emotions, highly arousing negative emotions, highly arousing illness-related words, and neutral words. Initially, they were asked to classify the words as pleasant or unpleasant, arousing or calming, large or small, with no explicit instructions for memorization. The free-recall task was unexpectedly conducted 45 minutes after the end of the learning phase. High-alexithymia individuals remembered significantly more illness-related words, and significantly fewer words related to negative emotions than low-alexithymia participants. Recall of illness words was positively correlated with the scores on each of the three subscales of the TAS-20.

The third study using a free-recall test^27^ examined the effect of alexithymia on the controlled retrieval of negative and neutral information in young and older adults, through a directed forgetting paradigm.^38^ In their experiments, after participants read a list of words, they were told to forget those words (which were “practice” words) and to read another list of words. After finishing reading the second list, participants were instructed to remember as many words as possible from the second list (remember list). The rationale behind this task was to probe cognitive control processes involved in memory by measuring the capacity of individuals to inhibit the retrieval of “undesired” memories (forget list). Thus, the goal here was to examine whether high-alexithymia individuals would exhibit greater difficulty inhibiting these unwanted memories (i.e., emotionally negative encoded items). Results showed that high-alexithymia individuals remembered more negative words from the first list (i.e., supposed to be forgotten) and fewer negative words from the second list (i.e., supposed to be remembered), compared to low-alexithymia individuals. This finding suggests that alexithymia is associated with a global decline in cognitive control over memory for emotional information, being related both to impaired intentional inhibition of negative emotional material, and to an impaired ability to recall “desired” emotional memories. These patterns were not present in memory for neutral materials.

In the studies discussed so far, high-alexithymia individuals performed worse than low-alexithymia individuals in basic recognition, remember/know, and free-recall tests, when they had to remember emotional information. In all these memory tests, participants encoded and remembered arbitrary stimuli. That is, they had to remember series of unrelated stimuli, which were typically not relevant to their personal lives. An interesting question regarding alexithymia and episodic memory is whether recollection of information of personal relevance is in any way affected by alexithymia. More specifically, whether alexithymia affects the recollection of memories of autobiographical relevance, namely, autobiographical memories.^39^

### Autobiographical memory tests

The question of whether autobiographical memory is affected by alexithymia was pursued by Lundh et al.,^40^ who examined the relationship between two measures: the TAS-20 and the Autobiographical Memory Test (AMT).^41^ The AMT is based on the presentation of some emotional words (positive and negative) and measuring the latency for the individual to provide the earliest autobiographical memory that comes to mind, relating to these words. There was no significant association between the TAS-20 and the AMT scores, only a slight trend toward positive correlation for the few participants exhibiting the highest levels of alexithymia (the sample was non-clinical). Thus, according to this study, the ability to remember personally relevant past events is not clearly affected by alexithymia.

Another study investigating alexithymia and autobiographical memory was reported by Muir et al.,^29^ and investigated whether different degrees of interoceptive awareness (sensitivity to internal bodily signals) had an impact on the Fading Affect Bias of autobiographical memory. The fading affect bias consists of greater forgetting for unpleasant than for pleasant autobiographical memories. This bias is thought to play a role in emotional regulation by helping individuals to maintain a positive view of the world and of themselves (see Walker et al.^42^, for a review). In the study reported by Muir et al.,^29^), alexithymia was measured by the TAS-20 and interoceptive awareness by the Multidimensional Assessment of Interoceptive Awareness (MAIA). Participants (13% of them within the clinical alexithymia range) were asked to recall pleasant and unpleasant events they had experienced in the last 12 months. The study showed that interoceptive awareness scores were positively associated with greater fading affect bias, as expected. The alexithymia level showed the opposite effect, being associated with a decreased fading affect bias. In general, pleasant affect faded more and unpleasant affect faded less as the level of alexithymia increased. When the alexithymia scores reached 61 or higher, there was no fading affect bias whatsoever, since pleasant and unpleasant affect faded at the same rate. These data suggest that, while higher interoceptive awareness is important for the preservation of pleasant autobiographical memories, high rates of alexithymia increase the forgetting of positive autobiographical memories.

In summary, what do these autobiographical memory experiments add to the current knowledge about alexithymia and episodic memory? The bias to retain more positive than negative autobiographical memories seems to be affected by alexithymia. In addition, interoceptive awareness seems to play an important role in this preferential long-term retention of positive memories. Caution should be exercised, however, when generalizing these data, since in one the reviewed experiments on this issue, no correlations between alexithymia and autobiographical memory were found.^40^

## DISCUSSION

Overall, the thirteen studies reviewed suggest that explicit memory for emotional information is reduced in high-alexithymia individuals, even in individuals with alexithymia levels below the clinical threshold. This reduction was noted not only when superficial processing tasks were employed (e.g., incidental learning in Suslow et al.,^36^ but also under deep processing conditions.^23^ The findings from the remember/know studies suggest that the recollection of emotional information is particularly impaired in individuals with high levels of alexithymia.^23^^,^ 24 These findings should be considered with caution, however, since the number of studies investigating these issues, and reviewed here, is relatively small. Thus, more research should be conducted to verify whether these findings are replicable.

Alexithymia is thought to be a multidimensional condition, with its dimensions represented by the subscales of the TAS-20.^1^^,^ 43 The association of alexithymia with episodic memory, therefore, perhaps results from impairments in two of these dimensions, represented specifically by the subscales “difficulties in identifying feelings” and “difficulties in describing feelings” of the TAS-20. We hypothesize here that these specific difficulties preclude thorough encoding of the emotional information of the to-be-remembered emotional items. Consequently, the typical advantage exhibited by individuals with average levels of alexithymia of learning and remembering emotional versus neutral information^44^ is not enjoyed by high-alexithymia individuals.

Interestingly, none of the studies reviewed found any impairment in memory for neutral information among high-alexithymia individuals, although this type of memory is usually weaker than memory for emotional information in the general population. In fact, results from the studies that found a significant difference regarding memory for neutral information showed that high-alexithymia individuals had an advantage over the low-alexithymia group in this aspect.^23^^,^ 27 ^,^ 37 Thus, the deficiencies found for individuals with higher scores of alexithymia cannot be attributed to a general memory deficit.

Furthermore, emotional memory deficits could not be attributed to other variables, such as anxiety, depression or effects of negative affect, since such potentially confounding factors were controlled for in most studies. Thus, the results of the studies reviewed suggest that the decline in memory for emotional materials in high-alexithymia individuals is more related to the processing of emotions, which is reduced in high-alexithymia individuals relative to the general population.

Seven of the reviewed studies involved non-clinical samples, with mean TAS-20 scores below the range of “possible alexithymia” (i.e., scores 51 or lower). Notably, despite this limitation, emotional memory reduction for alexithymia was observed in five of the studies (except for Lundh).^40^ In studies involving individuals with clinically relevant alexithymia, stronger effects were found.^23^^,^ 27 This raises the question whether the memory difficulties found here may influence treatment outcomes. As mentioned in the Introduction, alexithymia is associated with psychosomatic disorders, panic disorders,^12^ addictive behaviors,^14^ dissatisfaction in affective relationships,^13^ and gambling addiction.^15^ Because psychotherapy frequently require patients to become aware of their emotions, the difficulties of individuals with high alexithymia in describing and identifying their emotions, as well as in remembering the emotional aspects of events experienced, may represent a challenge for psychotherapists. Recent research, however, has shown that alexithymia is not reliably associated with treatment outcomes,^45^ although also see.^46^ Further research will be necessary to examine whether the specific memory deficits associated with alexithymia, suggested by the studies reviewed, are relevant for treatment outcomes.

Finally, the results of the implicit memory tests for emotional information were inconsistent. Two studies showed typical priming effects for emotional information. More specifically, Suslow^30^ showed a positive association between alexithymia and priming for emotional words, while Brandt et al.^28^ showed priming from emotional words when the targets were illness-related words. Two further studies, however, showed diminished priming effects for items preceded by angry faces,^25^ and “negative” priming effects for emotional words.^31^ Although more research will be necessary to confirm whether these inconsistencies are replicable, the current picture seems to suggest that the effect of alexithymia on implicit memory for emotional information is not particularly strong. Considering these findings in light of the data yielded by the remember/know and free recall experiments, one may hypothesize that the influence of alexithymia on memory for emotional information is more prominent over explicit memory processes, as recollective processes, specifically.

In summary, whereas it remains unclear whether implicit memory for emotional information is associated with alexithymia, explicit memory processes were shown to be impaired by high levels of alexithymia. This is especially evident in the studies using remember/know and free-recall tests. Nonetheless, because of the relatively small number of studies that have addressed this issue to date, further studies will be important in order to verify whether the current results are reproducible. Taking this limitation into account, the findings of the studies reviewed indicate that the difficulty of high-alexithymia individuals to perceive and describe their own feelings may cause a decrease in the typical memory advantage for emotional over neutral materials.

## References

[1] Sifneos PE (1973). The prevalence of 'alexithymic' characteristics in psychosomatic patients. Psychother Psychosom.

[2] Luminet O, Bagby RM, Taylor GJ (2001). An evaluation of the absolute and relative stability of alexithymia in patients with major depression. Psychother Psychosom.

[3] Taylor GJ, Bagby RM, Parker JDA (1997). Disorders of affect regulation: Alexithymia in medical and psychiatric illness.

[4] Luminet O, Rimé B, Bagby RM, Taylor GJ (2004). A multimodal investigation of emotional responding in alexithymia. Cogn Emot.

[5] Nielson KA, Meltzer MA (2009). Modulation of long-term memory by arousal in alexithymia: The role of interpretation. Conscious Cogn.

[6] Constantinou E, Panayiotou G, Theodorou M (2014). Emotion processing deficits in alexithymia and response to a depth of processing intervention. Biol Psychol.

[7] Wagner H, Lee V (2008). Alexithymia and individual differences in emotional expression. J Res Personal.

[8] Krystal H (1979). Alexithymia and psychotherapy. Am J Psychoth.

[9] Stone MA, Nielson KA (2001). Intact physiological response to arousal with impaired emotional recognition in alexithymia. Psychother Psychosom.

[10] Franz M, Popp K, Schaefer R, Sitte W, Schneider C (2008). Alexithymia in the German general population. Soc Psychiatry Psychiatr Epidemiol.

[11] Honkalampi K, Hintikka J, Tanskanen A, Lehtonen J, Viinamäki H (2000). Depression is strongly associated with alexithymia in the general population. J Psychos Res.

[12] Taylor GJ (2000). Recent developments in alexithymia theory and research. Can J Psychiatry.

[13] Humphreys TP, Wood LM, Parker JDA (2009). Alexithymia and satisfaction in intimate relationships. Pers Individ Dif.

[14] Pinard L, Negrete JC, Annable L, Audet N (1996). Alexithymia in substance abusers: persistence and correlates of variance. Am J Addict.

[15] Lumley MA, Roby KJ (1995). Alexithymia and pathological gambling. Psychother Psychosom.

[16] Borsci G, Boccardi M, Rossi R, Rossi G, Perez J, Bonetti M (2009). Alexithymia in healthy women: A brain morphology study. J Affect Disord.

[17] Bush G, Luu P, Posner MI (2000). Cognitive and emotional influences in anterior cingulate cortex. Trends Cogn Sci.

[18] Phan KL, Wagner T, Taylor SF, Liberzon I (2002). Functional neuroanatomy of emotion: a meta-analysis of emotion activation studies in PET and fMRI. Neuroimage.

[19] Lumley MA, Sielky K (2000). Alexithymia, gender, and hemispheric functioning. Compreh Psychiatry.

[20] Reker M, Ohrmann P, Rauch AV, Kugel H, Bauer J, Dannlowski U (2010). Individual differences in alexithymia and brain response to masked emotion faces. Cortex.

[21] McGaugh JL (2004). The amygdala modulates the consolidation of memories of emotionally arousing experiences. Annu Rev Neurosci.

[22] Taylor GJ, Bagby MR, Luminet O, Parker J.D.A., BAR-ON R. (2000). Assessment of Alexithymia: Self-Report And Observer-Rated Measures. The handbook of emotional intelligence.

[23] Luminet O, Vermeulen N, Demaret C, Bagby RM, Taylor GJ (2006). Alexithymia and levels of processing: Evidence for an overall deficit in remembering emotion words. J ResPers.

[24] Vermeulen N, Luminet O (2009). Alexithymia factors and memory performances for neutral and emotional words. Pers Individ Dif.

[25] Vermeulen N, Luminet O, Corneille O (2006). Alexithymia and the automatic processing of affective information: Evidence from the affective priming paradigm. Cogn Emot.

[26] Vermeulen N, Toussaint J, Luminet O (2010). The influence of alexithymia and music on the incidental memory for emotion words. Eur J Pers.

[27] Dressaire D, Stone CB, Nielson KA, Guerdoux E, Martin S, Brouillet D, Luminet O (2014). Alexithymia impairs the cognitive control of negative material while facilitating the recall of neutral material in both younger and older adults. Cogn Emot.

[28] Brandt L, Pintzinger NM, Tran US (2015). Abnormalities in automatic processing of illness-related stimuli in self-rated alexithymia. PLoS One.

[29] Muir K, Madill A, Brown C (2017). Individual differences in emotional processing and autobiographical memory: interoceptive awareness and alexithymia in the fading affect bias. Cogn Emot.

[30] Suslow T (1998). Alexithymia and automatic affective processing. Eur J Person.

[31] Suslow T, Junghanns K (2002). Impairments of emotion situation priming in alexithymia. Pers Individ Dif.

[32] Haviland MG, Warren WL, Riggs ML (2000). An Observer Scale to Measure Alexithymia. Psychosomatics.

[33] Donges U, Suslow T (2015). Alexithymia and memory for facial emotions. Universitas Psychologica.

[34] Yonelinas AP (2002). The nature of recollection and familiarity: a review of 30 years of research. J Mem Lang.

[35] Tulving E (1985). Memory and consciousness. Can Psychol.

[36] Suslow T, Kersting A, Arolt V (2003). Alexithymia and incidental learning of emotional words. Psychol Rep.

[37] Meltzer MA, Nielson KA (2010). Memory for emotionally provocative words in alexithymia: a role for stimulus relevance. Conscious Cogn.

[38] Anderson MC, Green C (2001). Supressing unwanted memories by executive control. Nature.

[39] Conway MA (2005). Memory and the self. J Mem Lang.

[40] Lundh LG, Johnsson A, Sundqvist K, Olsson H (2002). Alexithymia, memory of emotion, emotional awareness, and perfectionism. Emotion.

[41] Williams JM, Broadbent K (1986). Autobiographical memory in suicide attempters. J Abnorm Psychol.

[42] Walker WR, Skowronski JJ, Thompson CP (2003). Life is pleasant - and memory helps to keep it that way!. Rev Gen Psychol.

[43] Yoshida EMP (2007). Validade da versão em Português da Toronto Alexithymia Scale - TAS, em amostra clínica. Psicol Reflex Crit.

[44] Kensinger EA, Corkin S (2003). Memory enhancement for emotional words: Are emotional words more vividly remembered than neutral words?. Mem Cognit.

[45] De Vroege L, Emons WHM, Sijtsma K, van der Feltz-Cornelis CM (2018). Alexithymia has no clinically relevant association with outcome of multimodal treatment tailored to needs of patients suffering from somatic symptom and related disorders. A clinical prospective study. Front Psychiatry.

[46] Greenen R, Ooijen-van der Linden L, Lumley MA, Bijlsma JW, van Middendorp H (2012). The match-mismatch model of emotion processing styles and emotion regulation strategies in fibromyalgia. J Psychosom Res.

